# Platelet-Derived GARP Induces Peripheral Regulatory T Cells—Potential Impact on T Cell Suppression in Patients with Melanoma-Associated Thrombocytosis

**DOI:** 10.3390/cancers12123653

**Published:** 2020-12-05

**Authors:** Niklas Zimmer, Franziska K. Krebs, Sophia Zimmer, Heidrun Mitzel-Rink, Elena J. Kumm, Kerstin Jurk, Stephan Grabbe, Carmen Loquai, Andrea Tuettenberg

**Affiliations:** 1 Department of Dermatology, University Medical Center Mainz, 55131 Mainz, Germany; niklas.zimmer@unimedizin-mainz.de (N.Z.); franziska.krebs@unimedizin-mainz.de (F.K.K.); sophia.zimmer@gmx.net (S.Z.); heidrun.mitzel@unimedizin-mainz.de (H.M.-R.); stephan.grabbe@unimedizin-mainz.de (S.G.); carmen.loquai@unimedizin-mainz.de (C.L.); 2 Center for Thrombosis and Hemostasis (CTH), University Medical Center Mainz, 55131 Mainz, Germany; elena.kumm@unimedizin-mainz.de (E.J.K.); kerstin.jurk@uni-mainz.de (K.J.)

**Keywords:** GARP, platelets, Treg, melanoma, thrombocytosis

## Abstract

**Simple Summary:**

Thrombocytosis correlates with poor prognosis for treatment of malignant melanoma. Detailed information on how platelets modify the anti-tumoral immune response is still elusive. Analyzing the immunomodulatory capacities of platelets on huCD4^+^ T cells in vitro, we were able to show that platelets are able to induce regulatory T cells by the expression of glycoprotein A repetitions predominant (GARP), thus indicating a potential contribution to the immunosuppressive tumor microenvironment. Furthermore, we analyzed platelets of melanoma patients in stage I and IV. Melanoma patients with poor prognosis showed, besides an increased platelet count, a significant increase in GARP expression on platelets. This study suggests the contribution of platelets on the immune evasion in melanoma patients, opening a new potential way to target the immunosuppressive TME.

**Abstract:**

Platelets have been recently described as an important component of the innate and adaptive immunity through their interaction with immune cells. However, information on the platelet–T cell interaction in immune-mediated diseases remains limited. Glycoprotein A repetitions predominant (GARP) expressed on platelets and on activated regulatory T cells (Treg) is involved in the regulation of peripheral immune responses by modulating the bioavailability of transforming growth factor β (TGF-β). Soluble GARP (sGARP) exhibits strong regulatory and anti-inflammatory capacities both in vitro and in vivo, leading to the induction of peripheral Treg. Herein, we investigated the effect of platelet-derived GARP on the differentiation, phenotype, and function of T effector cells. CD4^+^CD25^−^ T cells cocultured with platelets upregulated FoxP3, the master transcription factor for Treg, were anergic, and were strongly suppressive. These effects were reversed by using a blocking anti-GARP antibody, indicating a dependency on GARP. Importantly, melanoma patients in different stages of disease showed a significant upregulation of GARP on the platelet surface, correlating to a reduced responsiveness to immunotherapy. In conclusion, our data indicate that platelets induce peripheral Treg via GARP. These findings might contribute to diseases such as cancer-associated thrombocytosis, wherein poor prognosis and metastasis are associated with high counts of circulating platelets.

## 1. Introduction

In cancer patients, one of the main reasons for tumor immune escape and therapy failure is the immunosuppressive tumor microenvironment. Herein, suppressive immune cells as well as inhibitory factors secreted by the tumor itself play a central role [1].

Whereas platelets are primarily known for initiating hemostasis by clotting at the site of injury, recent evidence indicates that they also influence tumor immune responses and the tumor microenvironment [2,3]. Cancer-associated thrombocytosis has been linked to the promotion of metastasis, invasion, tumor development, and consequently to poor clinical outcomes in various tumor entities [4]. Lower platelet activation statuses and counts in patient blood correlate with decreased metastasis [5,6]. This can be explained in further detail as platelets promote motility [7,8,9,10], epithelial–mesenchymal cell transition (EMT) [11], tumor cell survival [12,13,14], immune evasion [15], increased adhesion of tumor cells to the endothelium [16,17,18], and subsequent extravasation [11,19,20,21,22].

Platelet-derived transforming growth factor β (TGF-β) is an important immunomodulator with a multi-faceted role in the promotion of EMT and metastasis. It has been found to enhance metastasis in vivo by directly inducing the transition of cancer cells into a mesenchymal-like phenotype through the Smad and NFkB pathways [23]. Platelet-derived TGF-β also promotes metastasis indirectly through myeloid cells and fibroblasts by encouraging the remodeling of the extracellular matrix. Additionally, it is known to affect myeloid cells via TGF-β receptor II signaling, resulting in upregulation of TGF-β1, arginase, and inducible nitric oxide synthase (iNOS). Altogether, this leads to inhibition of effector cell functions, which further contributes to metastasis [24,25].

Once metastasized, tumor cells enter the bloodstream as circulating tumor cells (CTC), being faced with extreme shear stress and attack from immune cells. Herein, melanoma cells express chemokines, which attract and activate platelets (tumor cell induced platelet aggregation, TCIPA). Platelets adhere to tumor cells due to their expression of primary fibrinogen receptor αIIbβ3 integrin and P-selectin, which bind to CD44 and αvβ3 integrin on the surface of tumor cells [26,27]. These adhered platelets act as a protective shield for CTC against extreme sheer stress and facilitate and increase extravasation [28,29,30].

As mentioned above, CTC must also evade attack from immune cells in the blood stream in order to survive. One mechanism is by impairing the killing efficiency of natural killer (NK) cells. TGF-β, derived from activated and shielding platelets, antagonizes the interleukin-15 (IL-15) pathway that is responsible for NK cell proliferation and activation. In addition, it downregulates NKG2D, leading to a decreased natural killer (NK) cell efficacy. Collectively, this results in a decreased killing efficiency of NK cells and increased tumor cell survival [31,32,33]. It has also been shown that the attachment of platelets onto CTC leads to the transfer of platelet-derived surface molecules, such as MHCI, to the surface of CTC, effectively “disguising” CTC and thus preventing attack by NK cells [34].

GARP (glycoprotein A repetitions predominant) is encoded by leucine-rich repeat containing protein 32 gene (LRRC32) and represents a non-signaling docking receptor for latent TGF-β. The expression of GARP was initially described on the surface of platelets, but it has also been found on activated regulatory T cells (Treg) and on tumor cells, such as melanoma and glioblastoma [35,36,37]. GARP is recognized as playing an important role in the binding and release of TGF-β and hence in peripheral tolerance and progression of cancer.

In the past, our group defined an important role for GARP itself in the induction of peripheral tolerance [38]. GARP induces Treg with strong suppressive capacity and prevents chronic inflammatory disease in a humanized mouse model [37]. It also suppresses the effector function of CD8^+^ cytotoxic T cells and alternatively activates macrophages to favor tolerance induction [38]. Therefore, GARP represents a functionally relevant immunosuppressive molecule that contributes to peripheral tolerance by significantly preventing effector cell responses in the tumor microenvironment [36,38].

As described above, platelets exhibit important immunomodulatory functions, especially on T cells [39,40]. However, how platelets regulate T cell immunity is far from being completely understood. Given the fact that platelets express GARP on their surfaces and linking this to our previous work describing GARP as a key molecule in inducing peripheral tolerance, we hypothesized that platelets may be involved in the induction of peripheral tolerance and thus the promotion of malignancy and resistance to therapy by inhibiting host immunity. The present study investigated the effect of platelets on T effector cell function and clearly demonstrated that platelets led to the induction of Treg in a GARP-dependent manner. Melanoma patients showed significantly higher levels of GARP on the surface of platelets and an increase in platelet surface expression of the platelet activation marker CD62P (P-selectin). Late-stage melanoma patients with an overall increased frequency of platelets showed a decreased response rate to their applied immunotherapy.

Our data showed a possible contribution of platelets to the adaptive immunity, leading to a poor prognosis of cancer patients with cancer-associated thrombocytosis. Additionally, this opens up new possibilities to target platelets as a therapeutic option for the treatment of cancer.

## 2. Results

### 2.1. Expression of GARP on Platelets

**Figure 1:**
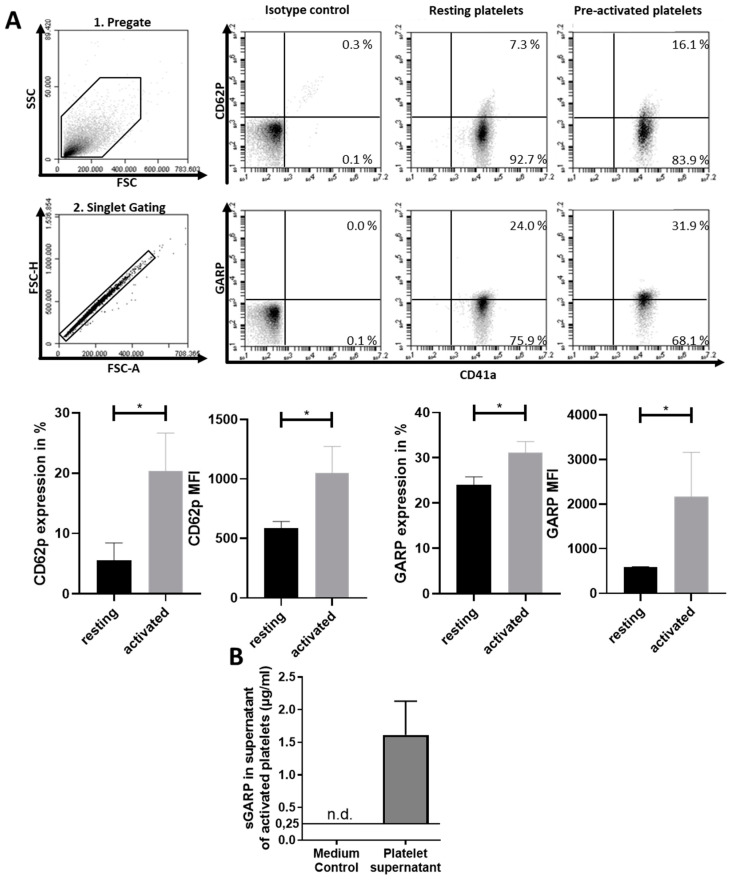
Glycoprotein A repetitions predominant (GARP) was expressed on the surface of platelets and was detectable in the supernatant of activated platelets. (**A**) Flow cytometric analysis of CD62P and GARP expression levels in resting and pre-activated platelets. Only singlets were used in the analysis. Isotype controls are shown. Bar diagrams of CD62P and GARP expression show pooled data of percentages (%) of positive cells and raw means (*n* = 3, means ± SD * *p* < 0.05, and n.s. determined by Student’s *t*-test). (**B**) Presence of soluble GARP (sGARP) in the supernatant of pre-activated platelets. sGARP content of the supernatant of 2 × 10^9^ activated platelets after 16 h compared to a negative medium control (n.d. = not detected). sGARP levels were determined by ELISA from three different healthy donors (HD).

**Figure A1:**
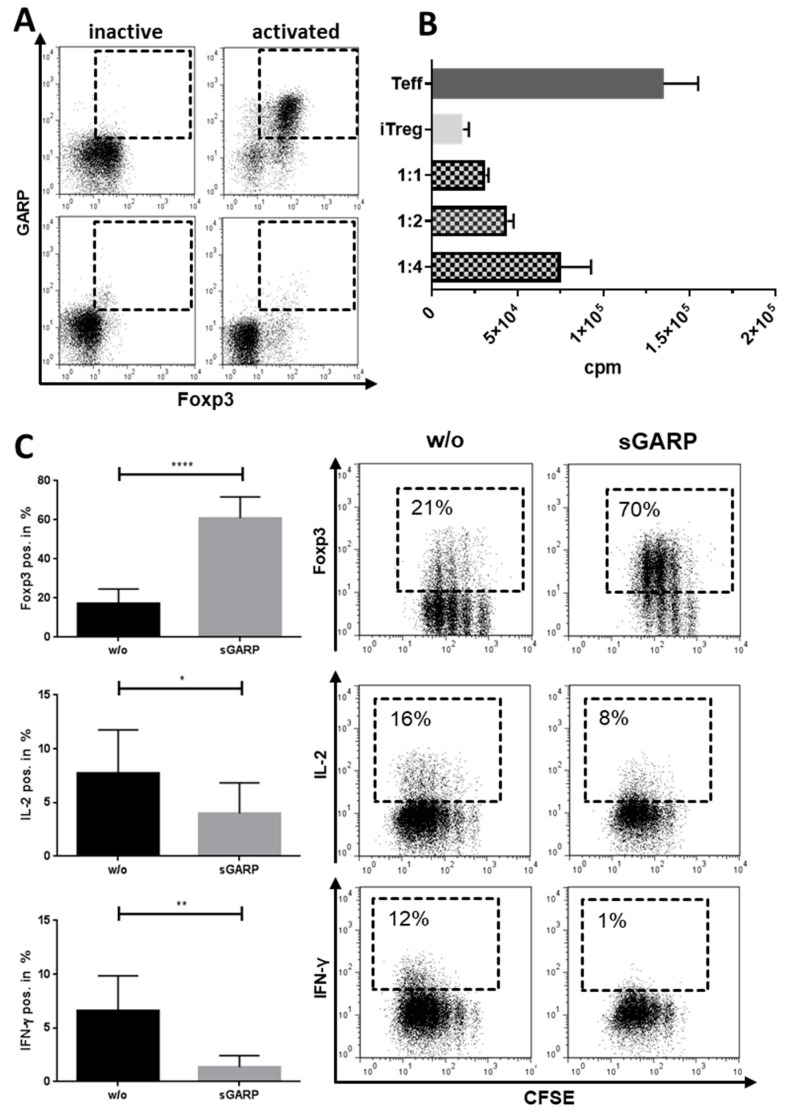
sGARP induced Treg. (**A**) GARP expression on Treg vs. Teff: GARP is an activation marker on Treg. 16h after stimulation, CD4^+^ CD25^+^ Treg showed a strong GARP expression compared to CD4^+^CD25^−^ Teff. One representative dot plot of four different experiments is shown. (**B**) Suppressive capacity of sGARP-conditioned T cells. CD4^+^CD25^−^ T cells were stimulated with 0.5 µg/mL anti-CD3 mAb and 1.0 µg/mL anti-CD28 mAb for 6 days in the presence of 10 µg/mL sGARP, resulting in Treg. Simultaneously, allogenic Teff were generated without the use of sGARP. After 6 days of stimulation, Teff and titrated Treg were cocultured and re-stimulated with anti-CD3 mAb and irradiated PBMC. After 4 days, proliferation was determined by tritiated thymidine (^3^H TdR) pulse. Results show the pooled data of three independent experiments. (**C**) sGARP induction of FoxP3, inhibition of proliferation, and inhibition of effector cytokine production in CD4^+^ T cells (*n* = 3, means ± SD, * *p* < 0.05, ** *p* ≤ 0.01, **** *p* ≤ 0.0001, and n.s. determined by one-way ANOVA).

GARP was initially described as being expressed on the surface of platelets [35]. Therefore, GARP expression was analyzed on resting and pre-activated platelets in combination with the platelet activation marker CD62P. In accordance with the literature, we detected GARP expression on resting platelets. Nevertheless, platelet activation led to a significant increase in the frequency of GARP^+^ platelets as well as the overall GARP expression (MFI) on platelets (Figure 1A). As we have demonstrated before, GARP can be shed from and found in the supernatants of either activated Treg or tumor cells, leading to the immunomodulation of T effector cells and macrophages (Figure A1). We investigated whether this process is also true for platelets. Pre-activated platelets were isolated from peripheral blood of healthy donors (HD) and cultured in X-VIVO 15, as described in the method section. Supernatants (platelet-conditioned medium, PCM) were collected after 16 h of culture and analyzed in an ELISA for GARP content. In all samples, analyzed GARP was detectable when compared to the medium control (X-VIVO-15) (Figure 1B). Importantly, no cellular contaminants (determined by microscopy and flow cytometry) were detectable.

### 2.2. Platelet-Derived GARP Induced Peripheral Regulatory T cells

**Figure 2:**
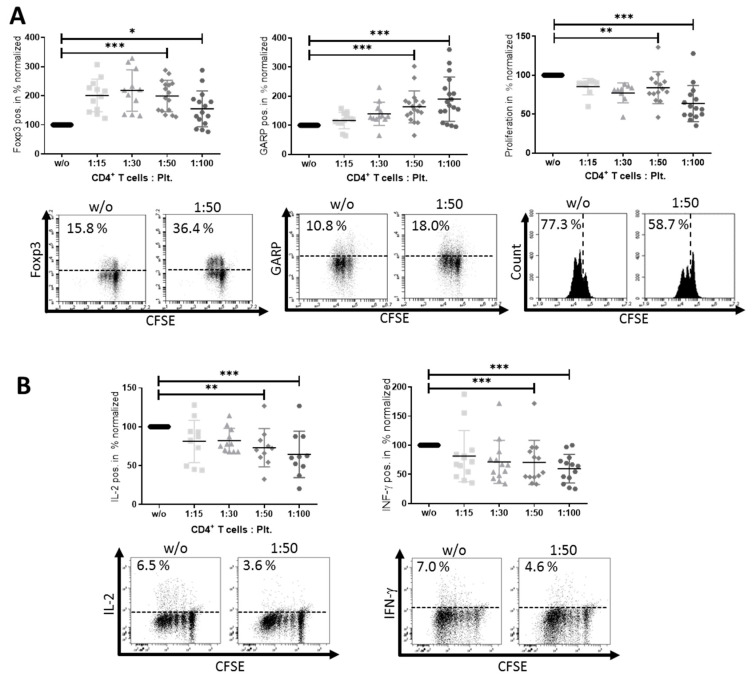
Platelets induce peripheral regulatory T cells (iTreg). (**A**) CD4^+^ CD25^−^ T cells and platelets were cocultured as indicated. Herein, carboxyfluorescein succinimidyl ester (CFSE)-labeled CD4^+^CD25^−^ T cells were stimulated with 0.5 µg/mL anti-CD3 mAb and 1.0 µg/mL anti-CD28 mAb with or without different ratios of platelets. The expression of Foxp3, GARP and the proliferation of cells were analyzed via flow cytometry on day 3 after stimulation. (**B**) Effector cytokine production of Interferon(IFN)-γ and interleukin (IL)-2 was analyzed using intracellular flow cytometry on day 6. Only live CD4^+^CD25^−^ T cells were included into the analysis. Representative dot plots of 12 independent experiments are shown. The graphs show cells cultured in the presence of platelets normalized to CD4^+^CD25^−^ T cells without platelets (expression levels were normalized to 100) (*n* = 12, means ± SD, * *p* < 0.05, ** *p* ≤ 0.01, *** *p* ≤ 0.001, and n.s. determined by Kruskal–Wallis test).

**Figure A2:**
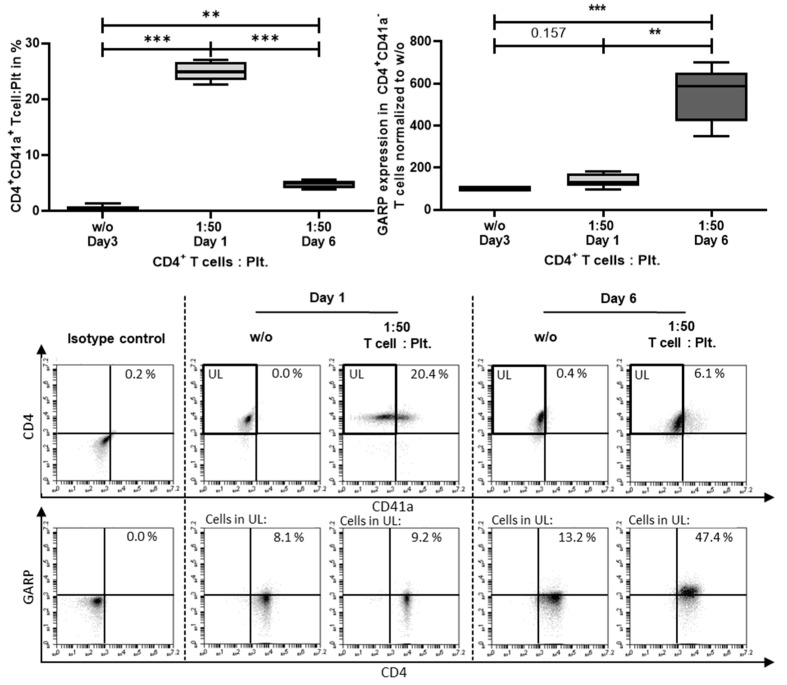
Platelets bound to T cells upon activation in coculture. CD4^+^CD25^−^ T cells were cocultured with or without platelets at the ratio of 1:50 and stimulated with 0.5 µg/mL anti-CD3 mAb and 1.0 µg/mL anti-CD28 mAb for 6 days. At day 1 and 6, platelet–T cell conjugates (CD41a^+^CD4^+^ double-positive cells) were analyzed via flow cytometry. For assessment of GARP expression, only CD4^+^CD41a- cells were included in the analysis (indicated by the pregating on the upper left (UL)). GARP expression was normalized to the untreated (w/o) control. Dot plots show one representative result of five independent experiments. Isotype controls are shown (*n* = 5, box and whiskers, medians + min/max, ** *p* ≤ 0.01, *** *p* ≤ 0.001, and n.s. determined by one-way ANOVA).

We next investigated the effect of platelet-derived GARP on peripheral blood CD4^+^CD25^−^ T cells. CD4^+^CD25^−^ T effector cells (Teff) and platelets were cocultured in different ratios, ranging from 1:15, 1:30, 1:50 to 1:100 Teff/platelets. With increasing platelet numbers, we detected a significant increase in Foxp3 and GARP expression on Teff (Figure 2A), whereas proliferation and effector cytokine production of Interleukin 2 (IL-2) and Interferon γ (IFN-γ) decreased (Figure 2B). To exclude the fact that the displayed GARP upregulation on Teff was due to contaminating adhering platelets on the Teff surface, we performed flow cytometry using a co-staining with anti-CD4 (Teff) and anti-CD41a (platelets) Abs. Within the first 24 h of coculture, about 25% of CD4^+^ T cells were also positive for the platelet marker CD41a, indicating adherence of platelets on the surface of Teff. This percentage significantly decreased within 6 days (CD4^+^CD41a^+^ 4.8%), as demonstrated by flow cytometry (Figure A2), showing that only a minor fraction of cells were CD4^+^CD41a^+^ double-positive. Notably, GARP expression on T cells increased over time. Because platelet effects on CD4^+^CD25^−^ T cells were most prominent at a ratio of 1:50, we used this ratio in the following experiments.

Having thus far used a non-canonical activation method, we next wanted to investigate a canonical, agonist-induced platelet activation, e.g., by thrombin being clinically more relevant. Therefore, we added 10 U/mL thrombin to the coculture. In comparison to the coculture without thrombin, this led to similar results, with increased Foxp3 and GARP expression and decreased cytokine production. Nevertheless, as described by Metelli et al. [41], thrombin leads to the cleavage of GARP on thrombocytes, which might partially explain the slightly reduced significances in the thrombin treated versus the 1:50 coculture control group (Figure A4). Therefore, we used TRAP-6, an additional canonical platelet activator, in our coculture. Here, we could again see similar results without any significant difference between TRAP-6-activated platelets (canonical activation) and the 1:50 coculture control group (non-canonical activation, Figure A5).

In sum, platelet-cocultured Teff displayed typical characteristics of induced peripheral Treg (iTreg), namely, reduced proliferation and effector cytokine production and increased FoxP3 expression.

**Figure 3:**
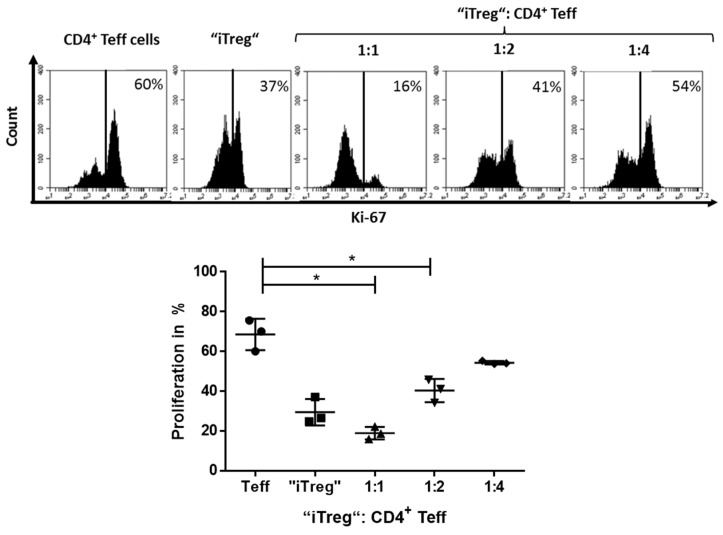
Platelet-induced iTreg suppressed T effector cells (Teff) cells. To analyze iTreg induction by platelets, we expanded CD4^+^CD25^−^ T cells for 6 days in the presence of platelets at the ratio of 1:50, as described previously, and were subsequently incubated at various ratios with 0.5 × 10^5^ CD4^+^CD25^−^ T cells and restimulated with 1 × 10^5^ irradiated peripheral blood mononuclear cells (PBMC) and 0.5 µg/mL anti-CD3 mAb. Proliferation was determined on day 3 of culture by Ki-67 staining (*n* = 3, means ± SD, * *p* < 0.05, and n.s. determined by one-way ANOVA).

**Figure A3:**
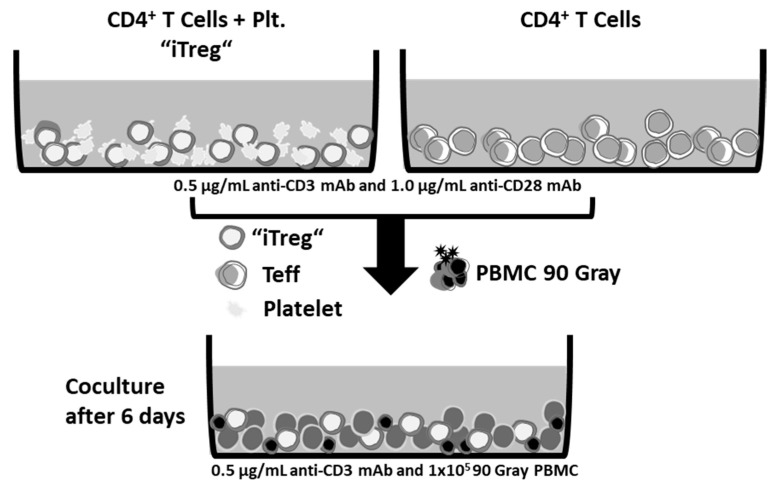
Experimental setup of a platelet-conditioned T cell suppression assay. 1 × 10^6^ CD4^+^CD25^−^ T cells were stimulated with 0.5 µg/mL anti-CD3 mAb and 1.0 µg/mL anti-CD28 mAb with or without 50 × 10^6^ platelets for 6 days. T cells cocultured with platelets developed an iTreg-like phenotype, whereas T cells cultured without platelets displayed a Teff phenotype. Next, iTreg and Teff were cultured at different ratios (iTreg/Teff: 1:1, 1:2, 1:4, and the corresponding controls) and restimulated with 0.5 µg/mL anti-CD3 mAb and 90Gy irradiated PBMC. Proliferation (Ki-67) was measured via flow cytometry 3 days after restimulation.

**Figure A4:**
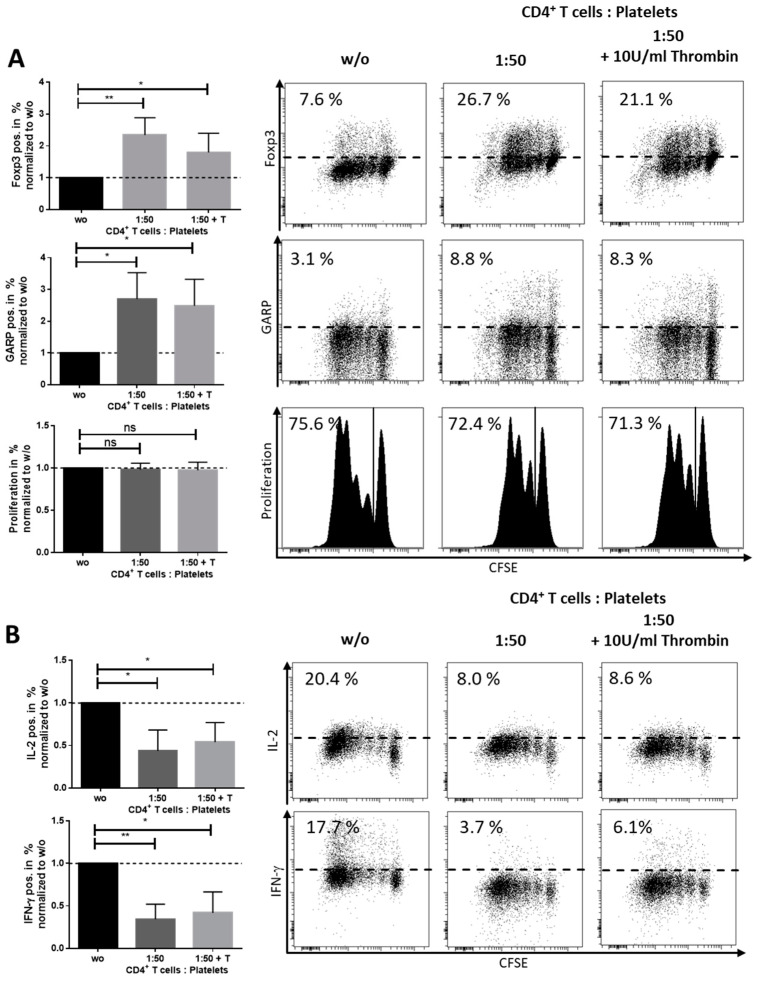
Thrombin-activated platelets induced a regulatory phenotype in CD4^+^CD25^−^ T cells. 1 × 10^6^ CD4^+^CD25^−^ T cells were stimulated with 0.5 µg/mL anti-CD3 mAb and 1.0 µg/mL anti-CD28 mAb with or without 50 × 10^6^ platelets for 6 days and treated with or without 10 U/mL thrombin. (**A**) Foxp3 and GARP expression and proliferation were determined at day 3 via flow cytometry. (**B**) Using intracellular flow cytometry, we analyzed cytokine production of IL-2 and IFN-γ on day 6. Dot plots show one representative result of five independent experiments (*n* = 5, box and whiskers, medians ± min/max, * *p* < 0.05, ** *p* ≤ 0.01, and n.s. determined by one-way ANOVA).

**Figure A5:**
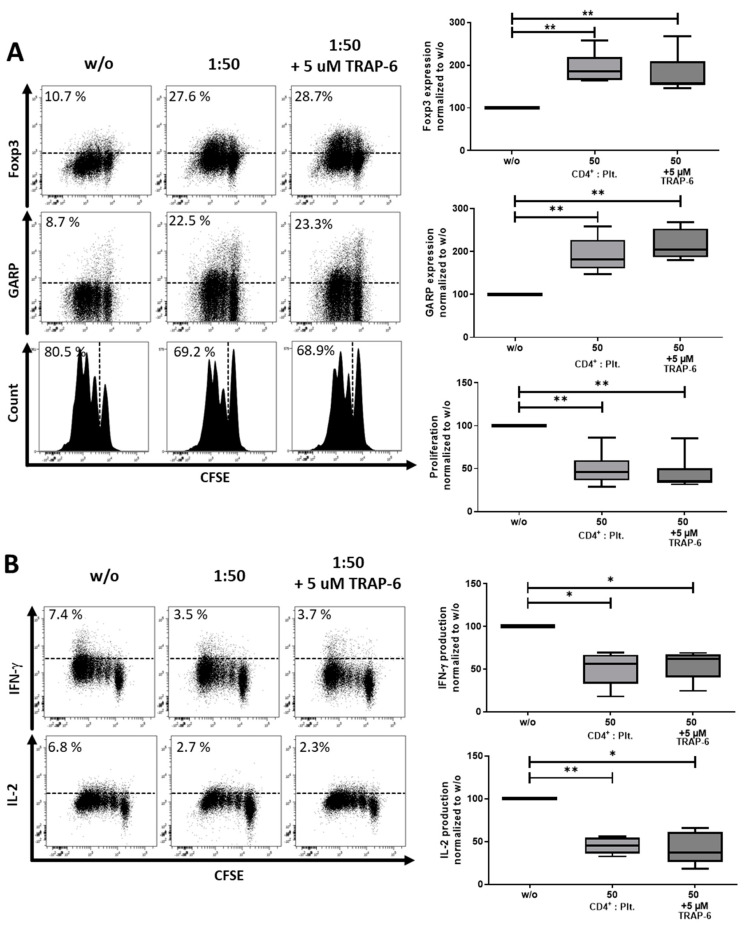
TRAP-6-activated platelets induced a regulatory phenotype in CD4^+^CD25^−^ T cells. 1 × 10^6^ CD4^+^CD25^−^ T cells were stimulated with 0.5 µg/mL anti-CD3 mAb and 1.0 µg/mL anti-CD28 mAb with or without 50 × 10^6^ platelets for 6 days and treated with or without 5 µM TRAP-6. (**A**) Foxp3 and GARP expression and proliferation were determined at day 3 via flow cytometry. (**B**) Using intracellular flow cytometry, we analyzed cytokine production of IL-2 and IFN-γ on day 6. Dot plots show one representative result of five independent experiments (*n* = 5, box and whiskers, medians ± min/max, * *p* < 0.05, ** *p* ≤ 0.01, and n.s. determined by one-way ANOVA).

To analyze whether these phenotypically altered anergic T cells resembling iTreg also had a suppressive function, we used them in a conventional suppressor assay. In detail, CD4^+^FoxP3^+^ iTreg (T cells pre-cultured with platelets for 6 days at the ratio 1:50) were harvested, washed, and then cultured together with untreated Teff (Figure A3) to investigate their suppressive function. Herein, platelet-induced Treg showed a significant suppressive capacity (Figure 3), as demonstrated by the reduced proliferation of T cells by Ki-67 staining in the suppression assay. Herein, decreasing numbers of platelet-induced Treg in the culture led to an increased proliferation of T cells, showing a dose-dependent suppression by the iTreg.

**Figure 4:**
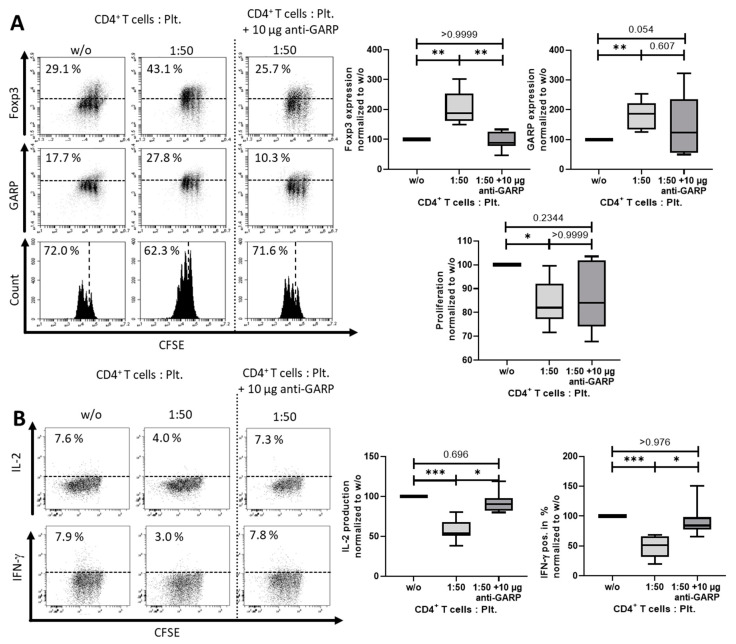
Platelets induced iTreg in a mainly GARP-dependent manner. (**A**) Blocking anti-GARP antibody inhibited platelet-induced effects on FoxP3 and GARP expression, as well as proliferation. CFSE-labeled CD4^+^CD25^−^ T cells were stimulated with 0.5 µg/mL anti-CD3 mAb and 1.0 µg/mL CD28 mAb with different ratios of platelets. In coculture, 10 µg/mL blocking anti-GARP Ab was added at day 0, as indicated. Foxp3 and GARP expression and proliferation is shown on day 3 after stimulation. (**B**) Cytokine production is shown on day 6. The graphs show cells cultured in the presence of platelets normalized to CD4^+^CD25^−^ T cells without platelets. For analysis, only live cells were included. Representative dot plots of eight independent experiments are shown (*n* = 9, box and whiskers, medians ± min/max, * *p* < 0.05, *** *p* ≤ 0.001, and n.s. determined by one-way ANOVA).

In order to determine whether the induction of iTreg is GARP-dependent, we added 10 µg/mL of a blocking anti-GARP Ab to the cocultures and again analyzed Foxp3 and GARP expression, proliferation, (Figure 4A) and cytokine production (Figure 4B). As demonstrated, addition of the blocking Ab led to a significant normalization of Foxp3 expression and restored the production of the effector cytokine IFN-γ to a normal level, indicating an induction of iTreg that was at least in part GARP-dependent.

### 2.3. Role of TGF-βand Platelet-Conditioned Medium in Platelet-Mediated iTreg Induction

**Figure 5:**
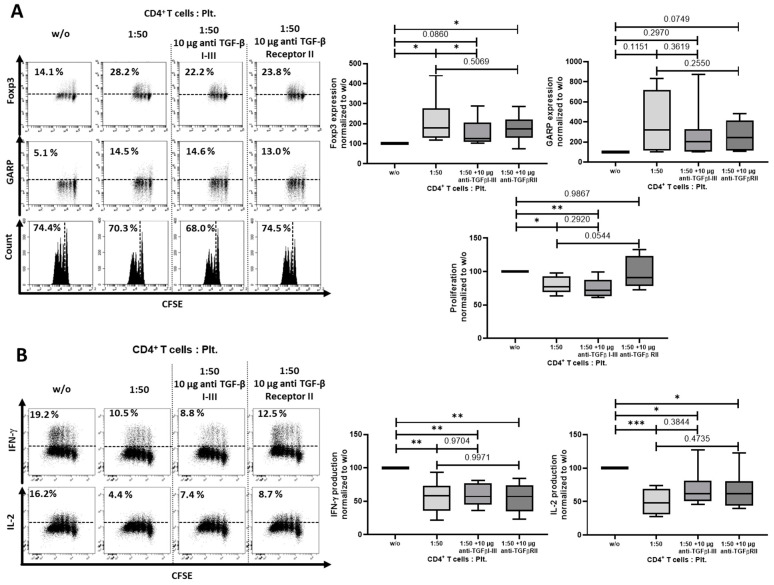
Blockade of transforming growth factor (TGF)-β I-III did in part prevent regulatory T cells (Treg) induction. (**A**) CFSE-labeled CD4^+^CD25^-^ T cells were cocultured with platelets in the ratio of 1:50 and were stimulated with anti-CD3 mAb (0.5 µg/mL) and anti-CD28 mAb (1.0 µg/mL) in the presence of either anti-TGF-β I-III (10 µg/mL) or anti-TGF-β receptor II (10 µg/mL) antibodies. Antibodies were added at day 0. The expression of Foxp3 and GARP and cell proliferation were determined on day 3 via flow cytometry. (**B**) Production of IL-2 and IFN-γ was assessed by intracellular flow cytometry on day 6. The graphs show cells cultured in the presence of platelets normalized to CD4^+^CD25^−^ T cells without platelets. Dot plots show one representative result of 10 independent experiments (*n* = 10, box and whiskers, medians ± min/max, * *p* < 0.05, ** *p* ≤ 0.01, *** *p* ≤ 0.001, and n.s. determined by one-way ANOVA).

GARP is known to be a surface docking receptor for TGF-β, and plays a dominant role in its activation and release. Thus, we next assessed the impact of TGF-β in the regulatory activity of platelet-derived GARP. Herein, the blockade of TGF-β signaling with blocking antibodies against TGF-β I-III showed a partial inhibition of the modulatory effects of platelet-derived GARP on Foxp3 regulation (Figure 5A) and IL-2 (Figure 5B) production, whereas proliferation and IFN-γ production was not affected. Using a blocking antibody against TGF-β receptor II (TGF-β RII), we had similar results, as Foxp3 and GARP were upregulated, whereas the production of IFN-γ and IL-2 were only partially inhibited. Only proliferation was strongly affected by the TGF-β RII blocking antibody, which was consequently restored to a normal level.

**Figure 6:**
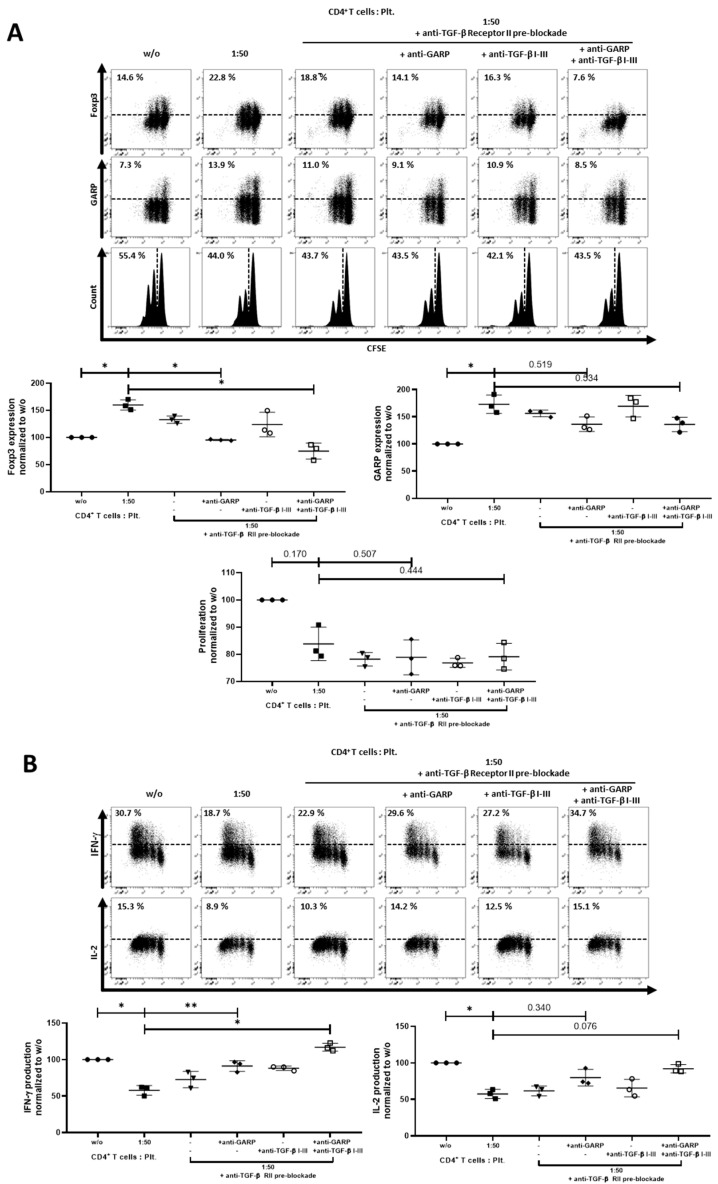
Combining blockade of TGF-β signaling and GARP led to a complete inhibition of platelet effects. (**A**) CFSE-labeled CD4^+^CD25^−^ T cells were cocultured with platelets in the ratio of 1:50 and were stimulated with anti-CD3 mAb (0.5 µg/mL) and anti-CD28 mAb (1.0 µg/mL). CD4^+^CD25^−^ T cells were incubated for 15 min with TGF-β receptor II (10 µg/mL) antibody prior to coculture, as indicated. Excess antibody was removed. Pre-treated CD4^+^CD25^−^ T cells were cultured in the presence of either anti-TGF-β I–III (10 µg/mL) and/or anti-GARP Ab (10 µg/mL) antibodies. Antibodies were added at day 0. The expression of Foxp3, GARP and cell proliferation were determined on day 3 via flow cytometry. (**B**) Production of IL-2 and IFN-γ was assessed by intracellular flow cytometry on day 6. The graphs show cells cultured in the presence of platelets normalized to CD4^+^CD25^−^ T cells without platelets. Dot plots show 1 representative result of 10 independent experiments (*n* = 3, means ± SD, * *p* < 0.05, ** *p* ≤ 0.01, and n.s. determined by one-way ANOVA).

To gain more insight into the relationship between TGF-β and GARP, we performed an additional experiment where the TGF-β receptor II was blocked on CD4^+^CD25^−^ T cells before coculture. The subsequent addition of either anti-TGF-β I-III Ab, anti-GARP Ab, or both only led to a complete inhibition of platelet effects in the samples with an anti-GARP Ab present. The combination of anti-TGF-β RII Ab, anti-TGF-β I-III Ab, and anti-GARP Ab showed the strongest effects on Foxp3, IL-2, and IFN-γ production, which were brought back to untreated levels. Anti-TGF-β RII Ab combined with anti-GARP Ab had the second strongest effect, followed by anti-TGF-β I-III Ab combined anti-GARP Ab and the use of only the anti-GARP Ab itself. The blockade of TGF-β alone by a combination of anti-TGF-β RII and anti-TGF-β I-III was not sufficient to inhibit platelet effects on T cells (Figure 6A,B).

These results demonstrate that the T cell modulating impact of platelet-derived GARP is in part but not completely associated with TGF-β signaling.

**Figure 7:**
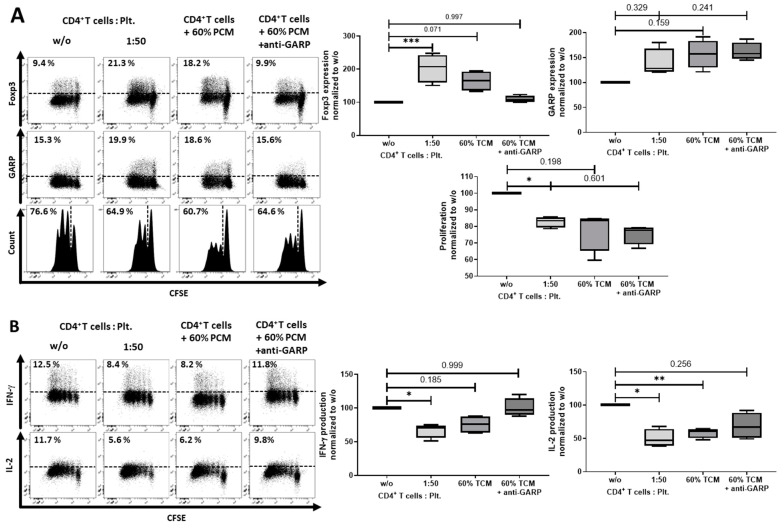
Platelet-conditioned medium (PCM) inhibited IFN-γ production, but failed to induce a Treg phenotype. (**A**) CD4^+^CD25^−^ T cells were cultured in X-Vivo 15 (Lonza, Basel, Switzerland) with 60% PCM content, with or without 10 µg/mL anti-GARP Ab and stimulated with 0.5 µg/mL anti-CD3 mAb and 1.0 µg/mL anti-CD28 mAb. Antibodies were added at day 0. The expression of Foxp3, GARP and cell proliferation were determined at day 3 with flow cytometry. (**B**) Cytokine production of IL-2 and IFN-γ was measured by intracellular flow cytometry on day 6. Dot plots show one representative result of five independent experiments (*n* = 5, box and whiskers, medians ± min/max, * *p* < 0.05, ** *p* ≤ 0.01, *** *p* ≤ 0.001, and n.s. determined by one-way ANOVA).

As shown in Figure 1B, soluble GARP (sGARP) was detected in PCM. To further assess the effects of PCM on the differentiation process of T cells, we cultured T cells with PCM. Addition of PCM to T cells resulted in a tendency of an upregulation of Foxp3, an inhibition of proliferation (Figure 7A), and a significantly lower production of the effector cytokine IFN-γ (Figure 7B). The addition of the blocking anti-GARP Ab lead to a normalization of the Foxp3 expression level and the IFN-γ and IL-2 production, whereas proliferation and GARP expression did not normalize.

### 2.4. Correlation of Thrombocytosis and Prognosis—Clinical Impact of Platelets Expressing Increased Levels of GARP

**Figure 8:**
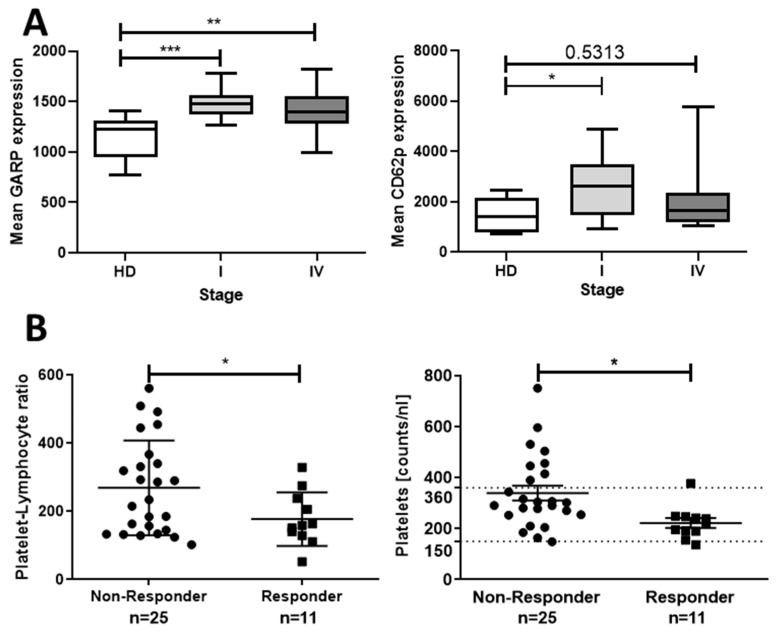
Increased platelet surface GARP levels in stage I/IV melanoma patient stage. (**A**) Comparison of platelet GARP and CD62P surface levels between HD (*n* = 8), stage I (*n* = 18), and stage IV (*n* = 17) melanoma patients assessed via flow cytometry. Means for GARP expression for HD = 1161, stage I = 1489, and stage IV = 1418. Means for CD62P expression are HD = 1470, stage I = 2611, and stage IV = 1977 (box and whiskers, medians ± min/max, * p < 0.05, ** p <.01, *** p <.001, and n.s. determined by unpaired *t*-test). (**B**) The platelet count in melanoma patients before starting immunotherapy using checkpoint inhibitors was measured as part of the routine blood tests. Platelet counts and the platelet–lymphocyte ratios of non-responders (*n* = 25) and responders (*n* = 11; staging results after 6 months of therapy) were compared. Means of platelet counts (PC) and the platelet–lymphocyte ratio (PLR) were as follows: progression: PC = 339.8, PLR = 269.0; stable disease: PC = 222.3, PLR = 177.5 (means ± SD, * *p* < 0.05, ** *p* ≤ 0.01, *** *p* ≤ 0.001, and n.s. determined by unpaired *t*-test).

By analyzing GARP expression on platelets from patients out of our first, initial cohort (*n* = 35) in both early and late-stage melanoma patients, we could detect increased GARP levels in comparison with HD controls. Nevertheless, GARP expression slightly differed between early stage I and late stage IV melanoma patients (Figure 8A). Additionally, platelets of stage I melanoma patients showed a stronger activation status compared to HD controls.

Several studies in different tumor entities indicate that cancer patients with thrombocytosis have poor prognoses [42]. In order to interpret these findings in the context of our own results, we analyzed a second, retrospective cohort of stage IV melanoma patients (*n* = 36) for their platelet counts and platelet–lymphocyte ratios (PLR) and correlated them to progression versus stable disease during therapy with checkpoint inhibitors (Figure 8B). Herein, non-responders to therapy with progressive disease showed significantly higher PLR and platelet counts than patients with stable disease, which responded to immunotherapeutic approaches.

## 3. Discussion

There has been an emerging role of platelets in the immunomodulation of cancer patients. Recent studies have indicated that platelets are present in the tumor microenvironment, and cancer-associated thrombocytosis has been linked to the promotion of metastasis, invasiveness, and tumor development and thus to poor clinical outcome in different tumor entities [4,43]. Tumors constantly activate the coagulation pathways, resulting in the generation of thrombin and consequently chronic platelet activation. Correlations between high platelet counts and shorter disease-specific survival are described for several tumor entities including lung, colon, breast, pancreatic, kidney, and gynecologic cancers. Platelets promote motility [8,9,10] and epithelial–mesenchymal cell transition, and readily bind to the surface of melanoma cells, thus protecting them from immunological clearance and/or chemotherapy-induced apoptosis [11].

The present study demonstrates a new and important role of platelets in mediating Teff inhibition by induction of Treg via a GARP-dependent mechanism. In agreement with the literature [44], GARP was found to be expressed on platelets to a certain extent and increased upon platelet activation, with a significant amount of GARP detected in the supernatants of activated platelets. This finding is of special interest for its implications on the tumor microenvironment as it is described that tumor cells lead to the chronic activation of platelets [45]. Coculture of platelets and Teff cells but also culture of Teff in the presence of PCM induced cells with a regulatory phenotype. This included upregulation of the Treg master regulator Foxp3, reduced proliferation, and decreased effector cytokine production, as well as induction of suppressive capacity. Our study did not exclude other platelet-derived factors that could mediate the described effects. Nevertheless, blocking experiments showed that a significant part of the immunomodulatory effects of platelets or PCM can be contributed to their GARP expression. The platelet numbers chosen for our coculture experiments resulted in a lower platelet/Teff-ratio (50:1) than usually present in peripheral blood (approximately 500:1). Despite this, GARP effects were already detectable at these lower platelet numbers, with no changes at higher-tested ratios (i.e., 100:1). Limitations in cell culture prevented detailed analysis of more physiological ratios at 500:1. In addition, the TGF-β signaling axis seemed to be at least in part associated to the investigated GARP effect in inducing iTreg, thus contributing to a rather inhibitory tumor micromilieu. In former studies, our group demonstrated that sGARP induced Treg through TGF-β receptor II (Figure A1) [37]. This is in agreement with recent studies. A deletion of GARP on platelets of mice showed a blunted TGF-β activity, which improved the CD4^+^ and CD8^+^ T cell immune response at the site of the tumor [46]. As described above, we could show that the observed effect by Rachidi et al. is not only TGF-β -dependent but also GARP dependent. The complete blockade of TGF-β signaling in combination with the blockade of GARP led to almost complete inhibition of platelet effects. This effect could not be achieved by the combination of anti-TGF-β I–III and anti-TGF-β receptor II alone, indicating that GARP plays an important role in the induction of Treg by platelets, which is in part independent of TGF-β signaling. This was supported by further studies, as deletion of GARP in mice led to an increase in CD4^+^ and CD8^+^ T cell anti-tumor activity. Another study analyzed the role of GARP in hemostasis and thrombosis. Herein, it was demonstrated that GARP-deficient mouse platelets showed normal activation responses in vitro. Moreover, megakaryocyte/platelet-specific GARP knock-out in mice did not affect the tail bleeding time or the occlusion time in the carotid and mesenteric arteries after FeCl3-induced thrombus formation, indicating no essential role of GARP in hemostasis and thrombosis [44].

Interestingly, the melanoma patients of the first cohort, independent of their tumor stage, expressed higher amounts of GARP on their platelets as well as exhibited higher platelet activation statuses. As described earlier, GARP is associated with inhibitory effects on immune cells in the tumor microenvironment. One could reasonably speculate then that GARP expression on platelets may differ between stage IV patients with poorer prognoses and stage I patients, as a further increase in GARP expression in stage IV patients could be expected. Nevertheless, a closer look into Figure 8b shows that there was a detectable minor shift of GARP expression levels from stage IV patients in comparison to stage I patients in the direction of HD GARP levels. This was supported by a decrease in significance of stage IV patients (*p*-value HD-stage I: 0.0005 ***) compared to stage I patients (*p*-value HD-stage IV: 0.0068 **). Whether this was due to response to immunotherapy (16 out of 17 patients of stage IV in our rather small first cohort were responders) must be analyzed in future studies with representative control groups. In several studies, including different tumor entities, such as ovarian, glioblastoma, head and neck, colorectal, and non-small cell lung cancer, it has been described that patients with thrombocytosis often have an advanced tumor stage, a higher tumor grade, and lower progression-free and median overall survival, and thus have a poorer prognosis. Collectively, this indicates thrombocytosis as an adverse prognostic factor in various tumor entities [47,48,49,50,51,52]. Our study (second cohort) is in agreement with these previous studies as it shows that reduced response to immunotherapy correlated to higher platelet counts/PLR.

Platelets may promote carcinogenesis in several ways. First, circulating tumor cells may use platelets as protective barriers in a complex system of evasion from the attack of immune cells, as well as possibly as mediators for attachment to endothelial cells when initiating extravasation at metastatic sites [16]. Furthermore, platelets have a role in the prevention of hemorrhage in newly formed tumor vasculature, which is structurally abnormal and lacks the stability of local resident vasculature [53]. Several cytokines and growth factors contained in the alpha granules of platelets, such as vascular endothelial growth factor (VEGF), epidermal growth factor (EGF), platelet-derived growth factor (PDGF), transforming growth factor β (TGF-β), interleukin 1β (IL-1β), IL-8, and CXC motif containing ligand 12 (CXCL12) may play diverse roles in the tumor micro-environment, including the promotion of invasion and metastasis through a positive regulation of the epithelial–mesenchymal transition (EMT) process and immune evasion [23,54,55].

The present study provides new insights into how platelets could promote tumor progression through the induction of Treg via GARP. A recent study by Metelli et al. (2020) showed that thrombin contributes to cancer immune evasion by proteolysis/cleavage of GARP and subsequent liberation of TGF-β. The authors proposed that blockade of GARP cleavage is a valuable therapeutic strategy to overcome resistance to immunotherapy. This paper supports our data, wherein we showed that GARP plays an important role in the inhibitory tumor microenvironment [41].

Nevertheless, we postulate that not only the soluble (cleaved) form of GARP but also the membrane-bound and thus contact-dependent GARP can mediate suppressive effects through induction of iTreg. In our study, addition of thrombin to the platelet–Teff coculture and thus potential GARP cleavage even slightly reduced the capacity of Treg induction by platelets. One possible explanation for this would be that thrombin-cleaved GARP is impaired in function and has decreased immunosuppressive capacity. Another explanation, which is supported by the weaker effect of PCM compared to platelet–Teff cocultures, could be that sGARP is less potent at inducing Treg in contrast to surface-expressed GARP. Using TRAP-6 as a platelet activator in this context did not lead to the possible cleavage of GARP from the surface of platelets, and therefore it had no impact on platelet effects on CD4^+^CD25^-^ T cells. Whether this effect can be attributed to a truncated form of GARP after thrombin cleavage or that sGARP has a decreased immunomodulatory capacity must be further investigated. In our hands, it was shown for the first time that this effect on tolerance induction via GARP could be due to the induction of T cells with a regulatory phenotype. This is of great importance as Treg play a major role in the suppression of anti-tumor immunity.

Platelets are the second most abundant cell type in peripheral blood and thus are easy to isolate, count, and analyze. Furthermore, they contain large amounts of TGF-β and are positive for GARP, which contributes to a large extent towards immune inhibition, as described in our work. Therefore, the analysis of platelets, including their frequency and GARP expression levels, could serve as predictive or prognostic biomarkers in relation to potential for immune evasion and reduced responses to immunotherapies and thus to poor prognosis. Certainly, this has to be verified in a future larger study. Another important implication that arises from our data is the concept of implementing platelet-modulating therapies within oncology and investing new therapeutic strategies that target platelets and/or GARP. For instance, this could be achieved with GARP-specific antibodies targeting different immune as well as tumor cells and platelets within the tumor microenvironment.

In conclusion, our study describes for the first time in the human system the mechanism of GARP-dependent, platelet-mediated T cell suppression. Our study linked platelets to immune inhibition and explained, at least in part, why cancer patients with cancer-associated thrombocytosis have poor prognoses. This makes platelets an additional attractive target in combinatorial cancer immunotherapy treatments, mainly through the development of new (antibody-based) anti-GARP therapeutic approaches. This strategy would not only target platelets but also activated Treg and GARP+ tumor cells, leading to the modulation of the inhibitory tumor microenvironment with the overall aim to overcome cancer’s resistance to immunotherapy through this combinatorial approach.

## 4. Materials and Methods

### 4.1. Isolation of Pre-Activated and Resting Platelets and Preparation of Platelet-Conditioned Medium

Pre-activated platelets: Blood bags were obtained from healthy donors (HD), with approval of the local ethical committee (Landesaerztekammer Rheinland-Pfalz, No.837.019.10 (7028)). Blood bags were transferred to conical tubes (Greiner bio-one, Kremsmünster, Austria #227261) and subsequently centrifuged for 15 min at 160× *g* at room temperature (RT). The resulting platelet-rich plasma (PRP) supernatant was collected and centrifuged for another 15 min at 200 × *g* at RT in order to deplete potential leukocyte contamination. Next, the PRP supernatant was washed with 1× Phosphate-buffered saline (PBS) at a ratio of 1:1 and centrifuged for 5 min at 2000 × *g* at RT. The resulting pellet containing platelets was resuspended, and platelets were counted and added in different ratios to the coculture experiments. These platelets had a higher CD62P expression as measured in flow cytometry compared to “resting platelets” (see below) and were therefore referred to as “pre-activated platelets”. Platelet-conditioned medium (PCM) was prepared by culturing 2 × 10^9^ pre-activated platelets for 16 h in 2 mL of cell culture medium at RT (X-Vivo 15, Lonza, Basel Switzerland, #BE02-060F), and, as a control, were either activated with 10 U/mL thrombin or TRAP-6, as indicated. Subsequently, platelets were centrifuged at 2000× *g* for 5 min at RT. The resulting supernatant was isolated and immediately used for analysis as well as for coculture experiments. The PCM was checked for cell residues by conventional microscopy and flow cytometry.

Resting platelets: In contrast, “resting platelets”, which showed a lower CD62P expression as measured in flow cytometry, were isolated at RT by first generating PRP. Citrated blood was supplemented with 2 mM EGTA and centrifuged for 10 min at 200× *g*. The resulting PRP was diluted with CGS buffer (120 mM NaCl, 12.9 mM trisodium citrate dihydrate, 30 mM d-glucose, pH 6.5) at a ratio of 1:1. For leukocyte depletion, the PRP was centrifuged for 10 min at 69× *g*. The resulting supernatant was subsequent centrifuged for 10 min at 400× *g*. The resulting pellet was resuspended in CGS buffer and again centrifuged for 10 min at 400× *g*. The platelets were resuspended in 1× PBS for flow cytometry [56].

### 4.2. Isolation and Stimulation of Human T Cell Populations

**Figure A6:**
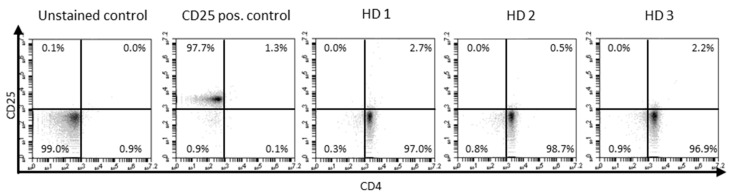
Flow cytometric analysis of T cell purity. CD4^+^CD25^−^ cells showed a purity of around 98% after isolation. CD4^+^CD25^−^ T cells were isolated as described in the manuscript. Shown are the unstained control, the CD25-positive staining control, and successful CD4^+^CD25^−^ isolations of three different healthy donors.

CD4^+^CD25^−^ T cells were isolated from buffy coat, as previously described [57]. The purity of the isolated CD4^+^CD25^−^T cells was around 98%, as checked by flow cytometry (Figure A6). For proliferation assays, cells were labeled with carboxyfluorescein succinimidyl ester (CFSE) and cultured in 48 well plates at 10^6^ cells/mL in X-Vivo 15 (Lonza, #BE02-060F, Basel, Switzerland). Percentage of proliferating cells was defined as cells with less CFSE signal than the initial signal strength of the unstimulated cells at the start of the assay. Cells were stimulated with 0.5 µg/mL anti-CD3 mAb (Clone OKT3) and 1 µg/mL anti-CD28 mAb (Clone 28.2, BD Pharmingen #555725, Heidelberg, Germany) in the presence or absence of different ratios of platelets and 10 µg/mL anti-GARP Ab (Origene AP17415PU-N, Rockland, MD, USA), 10 U/mL thrombin (Sigma-Aldrich #T7009-250UN, Munich, Germany), 10 µM TRAP-6 (H-Ser-Phe-Leu-Leu-Ag-Asn-OH trifluoroacetate salt (#4017752), Bachem Holding AG, Bubendorf, Switzerland), 10 µg/mL anti-TGF-β I-III (R&D Systems #MAB1835R), and anti-TGF-β receptor II (R&D Systems #AF-241-NA) at day 0, as indicated. For pre-blockade of TGF-β receptor II on CD4^+^CD25^−^ T cells, we incubated T cells with 10 µg/mL anti-TGF-β receptor II Ab for 15 min. The remaining unbound antibody was removed by washing cells with X-Vivo (Lonza, #BE02-060F, Basel, Switzerland)15 for 5 min at 400× *g*, twice. Alternatively, T cells were cocultured with PCM at the ratio indicated and proliferation was analyzed as described. For suppression assays, platelet-conditioned T cells (iTreg) were isolated from culture after 6 days, washed, and cocultured in a new assay with CD4^+^ T effector cells (Teff) for an additional 3 days at the ratio indicated. Cells were restimulated with 0.5 µg/mL anti-CD3 plus irradiated (90Gy) peripheral blood mononuclear cells PBMC. Proliferation was measured by Ki-67 staining via flow cytometry. Positive Ki-67 resembles proliferating cells instead of Ki-67 low cells.

### 4.3. Enzyme-Linked Immunosorbent Assay

Soluble GARP was analyzed by enzyme-linked immunosorbent assay (ELISA), according to the manufacturer’s protocol (R&D Systems #DY6055, Wiesbaden, Germany).

### 4.4. Flow Cytometry

**Figure A7:**
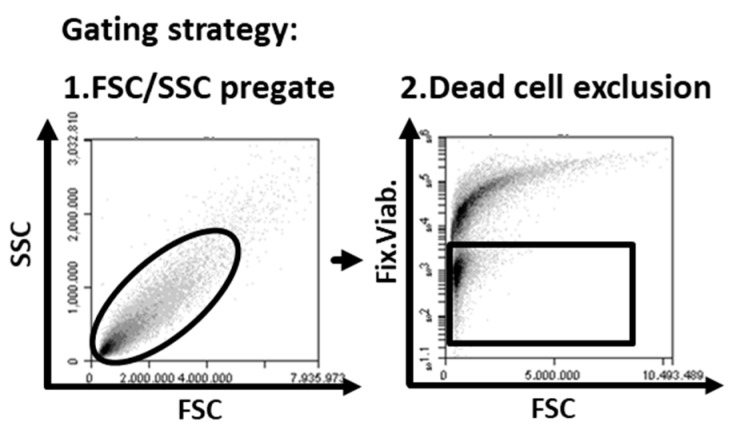
Gating strategy. Cells were pregated via forward- and side scatter (FSC/SSC) following a dead cell exclusion via fixable viability dye.

For flow cytometric analysis, cells were stained with fixable viability dye (Thermo Fisher #65-0865-14, Dreieich, Germany) prior to the antibody surface staining of anti-CD4 (BD Pharmingen #555348, Heidelberg, Germany), anti-CD41a (eBioscience #11-0419-42, Dreieich, Germany), anti-CD62P (ImmunoTools #21270624, Friesoythe, Germany), and anti-GARP (Miltenyi #130-103-820, Bergisch Gladbach, Germany). For intranuclear staining of FoxP3 or intracellular staining of IFN-γ and IL-2, cells were fixed and permeabilized with the Foxp3/Transcription Factor Staining Buffer Kit (eBioscience #00-5523-00, Dreieich, Germany) and subsequently stained with anti-Foxp3 (BioLegend #320208, San Diego, USA), anti-IL-2 (eBioscience #17-7049-42, Dreieich, Germany), and anti- IFN-γ (BD Biosciences #557643, Heidelberg, Germany). After 6 days of culture, T cells were harvested and expression of the cytokines IL-2 and IFN-γ were analyzed in T cells stimulated with 50 ng/mL phorbol 12-myristate 13-acetate (PMA; Sigma Aldrich #P1585-1MG, Munich, Germany) and 1 µg/mL ionomycin (Enzo Life Sciences #ALX-450-006-M001, Lörrach, Germany) in the presence of monensin (1.3 µM) (BD,#554724, Heidelberg, Germany) for 5 h. Cells were then permeabilized as described above and stained. Only live cells were included into the analysis (Figure A7). Flow cytometry was performed on an BD LSRII and BD Accuri C6 flow cytometer (BD Biosciences, Heidelberg, Germany) and analyzed using Cytobank software (Cytobank.org, Santa Clara, USA; [58] and the FACSVia Software (BD Biosciences, Heidelberg, Germany).

### 4.5. Patients

Patient samples were obtained from melanoma patients at different stages of disease after informed written consent. The study protocol (837.226.05 (4884)) was approved by the local ethics committee of Rhineland-Palatinate and Hessen (Landesärztekammer). All procedures in studies involving human participants were performed in accordance with the 1964 Declaration of Helsinki and its later amendments or comparable ethical standards. In the initial primary cohort, GARP expression on platelets and platelet activation status was measured from the blood of stage I (*n* = 18) and stage IV (*n* = 17) melanoma patients, of which 16 out of 17 patients responded to immunotherapy, by using flow cytometry. Therein, PRP was isolated as described, and platelets were stained for GARP and CD62P using flow cytometry. Next, platelet counts of the second retrospective cohort of stage IV melanoma patients undergoing checkpoint inhibitor treatment were analyzed in order to investigate platelet numbers, platelet–lymphocyte ratios, and therapy outcome in these patients (Table 1). Therefore, routine blood tests were performed during follow-up. Therapies included the checkpoint inhibitors ipilimumab (Yervoy, BMS, New York, USA), nivolumab (Opdivo, BMS, New York, USA), and pembrolizumab (Keytruda, MSD, Haar, Germany). Patients were divided into non-responders (progressive disease) and responders (complete response, partial response, or stable disease) according to Response Evaluation Criteria in Solid Tumors (RECIST) criteria in routine staging.

### 4.6. Statistics

Statistical analysis was performed using GraphPad Prism version 8.0.0 for Windows (GraphPad Software, San Diego, CA, USA, www.graphpad.com). Results were normalized to the untreated (*w/o*) samples as indicated. Bar diagrams and scatter plots display mean ± standard deviation (SD). Box and whiskers plots display median with the 25th and 75th percentiles and minimum to maximum whiskers. Statistical significance was determined using one-way ANOVA, Kruskal–Wallis test, and unpaired Student’s *t*-test, as indicated in the figure legends with * *p* < 0.05, ** *p* ≤ 0.01, *** *p* ≤ 0.001, and not significant. Not significant is indicated by *p*-values > 0.05.

## 5. Conclusions

Our data show, for the first time, that platelets are able to induce regulatory T cells in a GARP-dependent manner. Furthermore, melanoma patients with a high platelet count showed a reduced responsiveness to immunotherapy and a significantly increased expression of GARP on platelets. This could be of great importance, as thrombocytosis is associated with poor prognosis and metastasis in cancer. Our data implicate that the targeting of platelets in cancer could be a potential impactful new supplementary approach in addition to standard immunotherapy.

## Figures and Tables

**Table 1:** Patient characteristics.

**Cohort 1. Analysis of Platelet GARP and CD62p Expression Levels**	***n***	**%**
Patients	35	100
Stage I	18	51
Stage IV (responders)	17 (16)	49
**Cohort 2. Retrospective Analysis of Platelet Count and PLR**	***n***	**%**
Patients	39	100
Responder	11	28
Non-responder	25	64
Unknown outcome, lost to follow-up	3	8
